# The role of brain–liver–gut Axis in neurological disorders

**DOI:** 10.1093/burnst/tkaf011

**Published:** 2025-05-02

**Authors:** Li Pan, Lizheng Xie, Wenpei Yang, Shi Feng, Wenbao Mao, Lei Ye, Hongwei Cheng, Xiao Wu, Xiang Mao

**Affiliations:** Department of Neurosurgery, the First Affiliated Hospital of Anhui Medical University, No. 218 Jixi Road, Hefei, Anhui 230022, China; Department of Neurosurgery, the First Affiliated Hospital of Anhui Medical University, No. 218 Jixi Road, Hefei, Anhui 230022, China; Innovation and Entrepreneurship Laboratory for College Students, Anhui Medical University, No. 81 Meishan Road, Hefei, Anhui 230022, China; Department of Neurosurgery, the First Affiliated Hospital of Anhui Medical University, No. 218 Jixi Road, Hefei, Anhui 230022, China; Innovation and Entrepreneurship Laboratory for College Students, Anhui Medical University, No. 81 Meishan Road, Hefei, Anhui 230022, China; Department of Neurosurgery, the First Affiliated Hospital of Anhui Medical University, No. 218 Jixi Road, Hefei, Anhui 230022, China; Department of Neurosurgery, the First Affiliated Hospital of Anhui Medical University, No. 218 Jixi Road, Hefei, Anhui 230022, China; Department of Neurosurgery, the First Affiliated Hospital of Anhui Medical University, No. 218 Jixi Road, Hefei, Anhui 230022, China; Department of Neurosurgery, the First Affiliated Hospital of Anhui Medical University, No. 218 Jixi Road, Hefei, Anhui 230022, China; Department of Emergency, the First Affiliated Hospital of Anhui Medical University, No. 218 Jixi Road, Hefei, Anhui 230022, China; Department of Neurosurgery, the First Affiliated Hospital of Anhui Medical University, No. 218 Jixi Road, Hefei, Anhui 230022, China; Innovation and Entrepreneurship Laboratory for College Students, Anhui Medical University, No. 81 Meishan Road, Hefei, Anhui 230022, China

**Keywords:** Brain, Liver, Gut, Chronic traumatic encephalopathy, Depression

## Abstract

In recent years, with the increasing volume of related research, it has become apparent that the liver and gut play important roles in the pathogenesis of neurological disorders. Considering the interactions among the brain, liver, and gut, the brain–liver–gut axis has been proposed and gradually recognized. In this article, we summarized the complex network of interactions within the brain–liver–gut axis, encompassing the vagus nerve, barrier permeability, immunity and inflammation, the blood–brain barrier, gut microbial metabolites, the gut barrier, neurotoxic metabolites, and beta-amyloid (Aβ) metabolism. We also elaborated on the impact of the brain–liver–gut axis on various neurological disorders. Furthermore, we outline several therapies aimed at modulating the brain–liver–gut axis, including antibiotics, probiotics and prebiotics, fecal microbiota transplantation (FMT), vagus nerve stimulation (VNS), and dietary interventions. The focus is on elucidating possible mechanisms underlying neurological disorders pathogenesis and identifying effective treatments that are based on our understanding of the brain–liver–gut axis.

Highlights

- The brain, liver and gut can interact with each other by affecting the vagus nerve, blood–brain barrier, gut barrier and gut microbiota, which is called the brain–liver–gut axis.
- The brain–liver–gut axis significantly affects neurological disorders such as Parkinson’s disease, chronic traumatic encephalopathy, Alzheimer’s disease and depression.
- The brain–liver–gut axis can be regulated by fecal microbiota transplantation, dietary therapy, probiotics and prebiotics, antibiotics and vagus nerve stimulation to treat neurological disorders.

## Background

The influence of the liver and gut on neural function has gained recognition, with increasing research evidence indicating their connection to neurological impairments. The liver affects the nervous system, as observed in conditions such as liver cirrhosis and hepatic encephalopathy (HE), the underlying mechanisms of which, though not fully understood, are largely related to gut function and the microbiota. The gut microbiota itself is associated with several neurological disorders, such as Alzheimer’s disease (AD), chronic traumatic encephalopathy (CTE), Parkinson’s disease (PD) and depression [1–4]. Additionally, gut functionality affects the development of various diseases; for example, impaired gut barrier function can lead to microbial translocation and liver diseases, whereas peptides and hormones produced in the gut act on the vagus nerve and directly influence the brain. In turn, the brain regulates the liver and gut via the vagus and parasympathetic nerves. The liver, gut, and brain interact to form a pathway associated with many diseases, termed the brain–liver–gut axis. Researchers have already started exploring methods to regulate this axis, such as the use of sodium oligomannate to reshape the gut microbiota and mitigate neuroinflammation, thereby treating AD [5]. Study findings also suggest that probiotics and prebiotics can modulate the gut barrier and influence the vagus nerve, neurotransmitters, and blood–brain barrier (BBB) to treat nonalcoholic hepatitis, depression, and PD [6]. This review briefly examines the relationships among the brain, liver and gut, and discusses the mechanisms underlying that connect these vital systems while highlighting innovative therapeutic approaches and promising strategies aimed at enhancing long-term patient outcomes.

## Review

### The brain–liver–gut Axis

**Figure 1:**
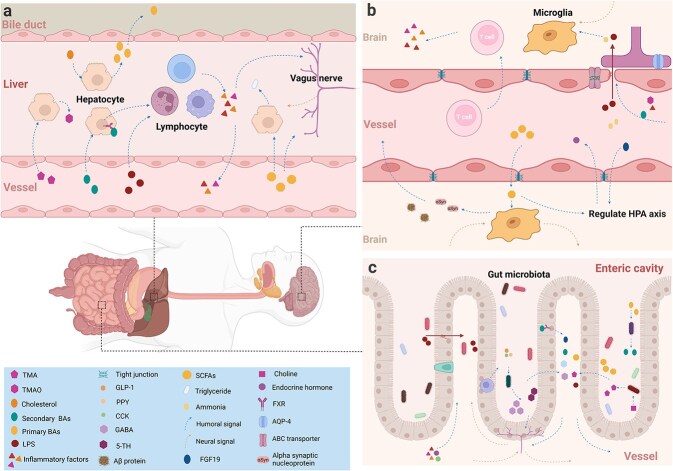
Schematic diagram of the brain–liver–gut axis interaction network. The brain, liver, and gut communicate with each other through complex neural, humoral, and immune signals. (a) this communication indicates that the brain–liver–gut axis can affect the metabolism of bile acids, TMAO, triglycerides, and other substances in the liver and thereby regulate liver inflammation. (b) the brain–liver–gut axis can affect the blood–brain barrier, brain hormone secretion, vagus nerve signaling, and chronic inflammation. (c) this ability indicates that the brain–liver–gut axis can affect the intestinal barrier, the secretion of intestinal hormones, the composition of the gut microbiota, and metabolism. TMA, trimethylamine; TMAO, trimethylamine N-oxide; BAs, bile acids; LPS, lipopolysaccharide; SCFAs, short-chain fatty acids; GLP-1, glucagon-like peptide-1; PYY, peptide YY; CCK, cholecystokinin; GABA, gamma-amino butyric acid; 5-HT, 5-hydroxytryptamine; FGF19, fibroblast growth factor 19. Created with BioRender.com

The brain is influenced by the liver and gut microbiota, with the three considered an interconnected whole. A classic example is HE, which originates from liver failure and manifests clinically as drowsiness, fatigue, depression, cognitive impairment, and even coma, with treatments such as lactulose and antibiotics targeting the gut and its microbiota. Clinical and animal model studies have demonstrated that, in the presence of hepatic cirrhosis—regardless of concomitant alcohol use—gut dysbiosis is significant and affects cognition and emotional behavior [7]. Clinical and laboratory evidence indicates the mutual influence among the nervous system, gut microbiota, and liver, suggesting that dysregulation in one aspect can impact the entire system. These mechanisms may involve the following factors (Figure 1).

### The Vagus nerve

The vagus nerve is one of the fastest pathway connecting the brain to the other organs. The receptors of the vagus nerve form different connections in the liver and gut. Furthermore, the multimodal receptors of these connections can respond to various mechanical, chemical, or hormonal signals [8]. The absence of afferent signals from the gut vagus nerve can cause various mental symptoms. In clinical case studies, vagotomy (a type of procedure used for treating peptic ulcers) has been reported to increase the incidence of mental health-related diseases [9]. In animal studies, mice with subdiaphragmatic vagal nerve afferent branch transections exhibit anxiety- and fear-related behaviors [10]. Recent research also revealed changes in c-Fos expression in vagal nerve afferent cell bodies after oral administration of *Campylobacter jejuni*, suggesting that gut bacteria can utilize the vagus nerve to alter the host’s mood and behavioral responses [11]. Furthermore, the vagus nerve can transmit biotic signals from the brain to the gut, prompting it to release hormones such as glucagon-like peptide-1 (GLP-1), Acetylcholine (ACh), peptide YY (PYY), interleukin −6(IL-6), tumor necrosis factor (TNF) and cholecystokinin (CCK) or inducing gut microbes to produce related metabolites such as short-chain fatty acids (SCFAs), lipopolysaccharide (LPS), hiscatecholamine, gamma-amino butyric acid (GABA), histamine and 5-hydroxytryptamine (5-HT) [12]. Between the gut microbiota and the host, there is a neural network called the enteric nervous system (ENS). The ENS reacts to signals from the gut microbiota and is largely responsible for coordinating gut function. The CNS communicates with the ENS via the vagus nerve, and the ENS can alter brain-regulated behaviors such as stress responses, anxiety, depression, social behavior, and cognition [13].

The nervous system of the liver is located near the portal vein, hepatic artery, and bile duct system, and includes two parts: sympathetic nervous system and vagus nerve [14]. The hepatic vagus nerve can sense the state of the liver and transmit neural signals to the brain, which then provides feedback to the parasympathetic nerves to regulate the liver fat content. For example, signals from the brain regulate very-low-density lipoprotein (VLDL) and triglyceride secretion via the vagus nerve and reduce liver fat content [15]. The systemic inflammation, depression-like behavior, and reduction in prefrontal synaptic protein levels caused by hepatic ischemia/reperfusion (HI/R) injury are also associated with the vagus nerve. Animal study findings have indicated that subdiaphragmatic vagotomy reversed these symptoms in mice with HI/R injury [16].

Recent studies have identified a complex brain-liver-gut neural reflex arc, indicating that the hepatic vagus nerve can regulate intestinal homeostasis. Specifically, hepatic vagus nerve afferent fibers indirectly sense the gut microenvironment and send signals to the brainstem nucleus of the solitary tract, which then sends signals to the parasympathetic vagus nerve and gut neurons, culminating in the induction and maintenance of the number of peripheral regulatory T cells (pTreg cells) in the gut [17]. Compared with nonvagotomized cirrhotic controls, vagotomized mice exhibit reduced levels of liver inflammation but increased levels of steatosis, increased brain-derived neurotrophic factor(BDNF) in the prefrontal cortex, and reduced glial cell activation [17].

These findings suggest that the vagus nerve is a bidirectional pathway that transmits signals along the brain-liver-gut axis, regulating gut ecology, liver microenvironment, and neuroinflammation.

### Immunity and inflammation

Neuroinflammation is closely related to changes in gut microbiota, currently, an increasing number of related mechanisms have been studied, with new evidence and therapeutic strategies being revealed. Animal experiments have revealed that destruction of gut microenvironment in mice with brain injury leads to the migration of gut T lymphocytes to damaged brain tissue, triggering CNS inflammation [18]. Additionally, changes of the gut microbiota can lead to increased metabolism of phenylalanine and isoleucine peripherally, cause the accumulation of circulating T-helper 1 (Th1) cells. Accumulation of circulating Th1 cells contribute to the activation of brain M1 microglia, thereby causing neuroinflammation relate to Alzheimer’s disease. GV-971, a sodium oligomannate, can regulate gut microbiota homeostasis and reduce the accumulation of pathogenic metabolites, control inflammation, and reverse cognitive impairment. Alterations in neuroendocrine factors can also cause gut inflammation. For example, during stress such as trauma, increased corticotropin-releasing hormone levels lead to elevated corticosterone levels, which promote the maturation of mast cells in the intestinal mucosa, thereby inducing neurogenic intestinal inflammation [19].

As the central organ for systemic metabolism and immune regulation, liver dysfunction can lead to the accumulation of toxic substances and an increase in inflammatory factors, some of which can even cross the BBB, causing CNS inflammation. For example, liver dysfunction can lead to manganese deposition, and neuroinflammation is associated with the neurotoxic effects of manganese [20]. Studies have shown that manganese can regulate microglial inflammatory cytokine output through the nuclear factor-κB pathway and induce microglia to release hydrogen peroxide, leading to neuroinflammation [21, 22]. Additionally, the beta-amyloid (Aβ) protein is mainly metabolized in the liver, and liver dysfunction can cause Aβ protein metabolism disorders, leading to neuroinflammation and AD progression [23]. The liver itself can produce inflammatory factors, such as IL-6 and adiponectin (APN). IL-6, an important inflammatory factor produced by hepatocytes and leukocytes, can affect brain function via the liver–brain axis. For example, in bile duct ligation-induced disease behavior model mice, liver and peripheral IL-6 levels are significantly elevated. The liver expresses the IL-6 receptor (IL-6R), which binds IL-6. The IL-6/IL-6R complex then interacts with transmembrane glycol proteins on brain endothelial cells, initiating signal transduction cascades [24]. IL-6-deficient mice exhibit significantly reduced disease behavior [25]. APN, a cytokine primarily expressed in hepatic adipocytes, has the function of regulating neuroinflammation. Study findings have suggested that circulating trimeric APN can cross the BBB, and regulates microglial-mediated neuroinflammation [26].

The liver and gut communicate directionally due to the bile duct, portal vein, systemic circulation, and vagus nerve, with their inflammation being mutually causal and influential. The liver plays a significant role in the inflammatory bowel disease (IBD) via the intricate gut-liver axis [27, 28]. Bile acids(BAs), as a medium of communication between the liver and gut, significantly affect the progression of colorectal diseases [29]. A study in 2024 showed that the liver can sense inflammatory factors from the gut, and regulating the proliferation of intestinal stem cells (ISC) to maintain gut homeostasis. Specifically, the liver releases pigment epithelium derived factor (PEDF), which can inhibit ISC proliferation by suppressing the Wnt/β—catenin signaling pathway. When the liver senses inflammatory signals from the intestine, the release of PEDF decreasesaph, thereby accelerating ISC proliferation and promoting intestinal regeneration. [30]. Moreover, a close relationship between chronic liver disease (CLD) and IBD has been reported. Intestinal inflammation will disrupt the integrity of the gut barrier, leading to the production of metabolism-associated molecular patterns (MAMPs) by the gut microbiota entering the liver, which induce liver inflammation and promote fibrosis progression [31–33]. Immunoglobulin A (IgA), produced by the liver, gut B cells, and plasma cells, is a crucial component of gut mucosal immunity. When liver damage or other factors cause IgA deficiency, persistent dysbiosis can result, thereby leading to gut inflammation and metabolic disorders [34].

### The blood–brain barrier

Many liver disease patients have an impaired BBB, which allows cytotoxic molecules such as ammonia and lactate to enter the brain, leading to increased white matter water content and edema [35]. Aquaporin-4 (AQP-4) is most commonly found on the surface of astrocytes in the brain and is a bidirectional transmembrane aquaporin, its main function in the brain is to regulate water homeostasis [36]. AQP-4 is related to BBB permeability and the development of brain edema. Studies have shown that various liver diseases can lead to the dysregulation of AQP-4 [37]. Autopsy analysis of frontal cortex tissues from patients with acute liver failure revealed elevated AQP-4 levels in astrocyte endfeet [37]. It was observed that in the brains of HE mice, the level of AQP-4 increased, while the expression of tight junction (TJ) protein decreases in the same brain regions [38]. Additionally, cirrhosis can increase BBB permeability by disrupting ATP-binding cassette (ABC) transporters. On the other hand, increased BA levels contribute to BBB opening. Excess BA levels can affect BBB integrity by altering the structure of TJs, leading to increased BBB permeability in bile duct-ligated HE model animals [39].

The gut can influence the BBB in many ways, including through gut microbes and their metabolites, various cytokines secreted by the intestinal epithelium, and neural signals originating from the gut. Studies have shown that gut microbes and their metabolites can regulate the expression of TJ proteins such as occludin and ZO-1 in the brain, thereby disrupting the integrity of the blood–brain barrier [40], absence of TJ proteins resulted in increased levels of inflammatory factors, further exacerbating blood–brain barrier damage and forming a vicious cycle [41]. Additionally, when the gut barrier is damaged or liver function is abnormal, pathogenic gut bacteria and their metabolites, LPS, can infiltrate the bloodstream. This infiltration leads to endotoxemia and triggers systemic inflammation. Consequently, microglia are activated, releasing inflammatory mediators like interleukin-1β (IL-1β) and tumor necrosis factor-alpha (TNF-α). These mediators further exacerbate the permeability of the blood–brain barrier, creating a cascade of detrimental effects. [42, 43].

### The gut barrier

In general, the gut barrier is composed of intestinal epithelial cells and junctional proteins. The intestinal epithelium continuously renews itself every 4–5 days to maintain gut barrier integrity. Junction proteins include TJ proteins as well as scaffold proteins [44, 45]. The liver and gut are closely connected in many ways. Damage to the gut barrier can cause liver dysfunction, and liver dysfunction can also damage the gut barrier.. When there is gut dysbiosis, which reduces the abundance of probiotics such as Lactobacillus, Bacteroides, and Bifidobacteria, TJ proteins are damaged and change gut permeability, leading to the entry of many bacteria and toxic substances into the blood, thereby causing liver injury [46]. For example, LPS, a pathogen-associated molecular pattern, usually does not enter the bloodstream, but while integrity of the gut barrier is compromised, LPS can enter the portal vein by combining with LPS-binding protein (LBP), thereby activating hepatic inflammasomes and ultimately leading to liver inflammation and fibrosis [47, 48]. Natural killer cells constitute the main lymphocyte population in the liver. When abnormally activated, they release excessive amounts of immunomodulatory cytokines, including IFNγ, IL-4, and IL-13, causing gut barrier disruption [49].

Many clinical studies have shown that traumatic brain injury (TBI) can lead to gut barrier dysfunction [50]. Laboratory evidence indicates that three hours after TBI, intestinal epithelial cell shedding, villus edema, and stromal edema occur, and these changes progress over time in the controlled TBI (c-TBI) model. The underlying mechanism is speculated to be related to decreased claudin-1 expression and increased activation of enteric glial cells (EGCs) [51, 52]. In addition to increasing the load on the liver, damage to the gut barrier can also cause CNS inflammation and degenerative changes, mainly due to the entry of toxic substances such as LPS into the blood. Clinical trials have demonstrated that peripheral injection of LPS in healthy adults strongly activates microglia [53]. Serum LPS levels correlate with gut barrier permeability in AD and PD patients, and circulating LPS levels are closely related to the severity of the disease.

### Gut microbe metabolites

#### Short-chain fatty acids

The guts of healthy adults contain microbes that produce approximately 50–100 mM of SCFAs, including acetate, propionate, and butyrate, daily [54]. SCFAs have three primary functions in the liver and gut: providing an energy source, serving as substrates for reactions, and acting as signaling molecules [55]. As an energy source, butyrate can provide energy for colon epithelial cells and is the most important SCFA for maintaining colon health; As a reaction substrate, acetate is absorbed from the colon into the liver and serves as a substrate for cholesterol synthesis; As a raw material, Propionic acid participates in gluconeogenesis, protein synthesis, and fatty acid synthesis in the liver; As a signaling molecule, SCFA can induce liver cells and adipocytes to release Fasting-induced adipocyte factor, thereby reducing the accumulation of triglycerides [56]. However, the effects of SCFAs are not entirely beneficial [57]. A recent study has shown that excessive butyrate in the circulation is closely related to bile stasis and jaundice, ultimately resulting in jaundice-type hepatocellular carcinoma (HCC) [58].

As metabolites of gut microbes, some SCFAs can cross the BBB after entering the peripheral circulation [59]. SFCAs can function as signaling molecules to regulate physiological functions and play immune-modulatory roles to regulate neuroinflammation [60]. As signaling molecules, SCFA can directly act on the hypothalamus, stimulate the hypothalamic pituitary adrenal (HPA) axis, and indirectly affect the central nervous system. For example, SCFAs act on fatty acid receptors or taste receptors, regulating gut motility and secretion [61]. Additionally, SCFAs entering the brain can reduce LPS related neuroinflammation in the hippocampus [62].

#### Bile acids

Hepatocytes produce primary BAs. These primary BAs then enter the gut through the biliary system, where gut bacteria convert them into secondary BAs [63]. BAs help digest dietary fats, cholesterol, and fat-soluble vitamins and act as signaling molecules by stimulating membrane receptor G protein coupled receptor 5 (TGR5) and farnesoid X receptor (FXR) to regulate energy metabolism [64]. BAs can promote FXR expression in the ileum, and elevated FXR expression serves as a negative feedback signal to reduce the expression of apical sodium dependent bile acid transporters in intestinal epithelial cells, thus regulating BA secretion and maintaining mucosal homeostasis in the gut [65]. In the liver, FXR plays a pivotal role by enhancing the expression of transport proteins of choline and cholesterol, while simultaneously suppressing the expression of NF-κB [66], thereby maintaining hepatic homeostasis. Overall, BA and FXR interactions regulate bile secretion and inflammation, thus inhibiting liver disease progression [67]. TGR5 can be activated by BAs to perform multiple functions, such as regulating bile secretion, improving pancreatic function and insulin sensitivity, and modulating liver Treg and Th17 signaling activity [68].

BAs significantly influence the brain beyond regulating inflammation and energy metabolism in the liver and gut. Studies have shown that patients with IBD have secondary BA deficiencies due to gut dysbiosis, which affects ZO-1 and occluding function in the brain, thereby increasing BBB permeability [69] and increasing the levels of CNS inflammatory mediators. Supplementing secondary BAs by activating TGR5 can reverse CNS inflammation [70]. Additionally, BAs stimulate FXR expression in the ileum, leading to an increase in fibroblast growth factor 19 (FGF19), it can cross the BBB and act on the hypothalamus to regulate glucose homeostasis and HPA axis function [71, 72]. Research also indicates that changes in BA levels may be associated with PD and AD [73, 74].

#### Choline

Choline has multiple sources and can be directly absorbed from food or produced endogenously in the liver. It is a component of the cell membrane and is essential for the formation of phosphatidylcholine in VLDL. Choline deficiency can lead to lipid peroxidation in hepatocytes, causing liver damage [75]. Gut microbes influence the body’s choline levels and participate in choline metabolism. Some gut bacteria can convert choline into dimethylamine or trimethylamine (TMA). Once formed, TMA can be transported to the liver, where it undergoes further oxidation to produce trimethylamine N-oxide (TMAO). The accumulation of TMAO associated with liver damage caused by increased triglyceride accumulation, leading to hepatic steatosis [76]. Furthermore, animal experiments have shown that TMAO can accelerate brain aging and lead to cognitive impairment [77, 78]. Additionally, TMAO metabolism disorders due to gut microbiota dysbiosis directly affect the BBB and influence cerebrovascular and neurological health [77].

### Related diseases

#### Alzheimer’s disease

Alzheimer’s disease is the leading cause of dementia, with diagnosis primarily relying on clinical symptoms and the detection of amyloid-beta (Aβ) and phosphorylated tau proteins [78]. Research suggests that, before cognitive impairment is observed, changes in neurons, microglia, and astrocytes drive the underlying progression of the disease [79]. Neuroinflammation [80], abnormal BBB permeability [81], and immune system dysfunction [82] lead to the accumulation of Aβ peptides, which induce further tau protein phosphorylation [83], causing neuronal necrosis and disease progression. In addition to the core markers of AD— Aβ, tau proteins, and microglial responses—it is evident that the vascular system, BBB, brain clearance system, peripheral immune system, and potentially gut microbes also affect the clinical progression of the disease [84, 85]. The liver holds a pivotal role in AD, not only by influencing neuroinflammation but also by participating in Aβ clearance [23]. Recent studies have revealed that the liver, in addition to clearing Aβ peptides peripherally, can also bidirectionally regulate plasma 14,15-epoxyeicosatrienoic acid (14,15-EET) levels by modulating liver soluble epoxide hydrolase (sEH) activity, with 14,15-EET crossing the BBB and regulating Aβ metabolism [86]. Animal experiments have shown that clearing the guts of mice of microbes can reduce brain Aβ accumulation and neuroinflammation in AD model mice, while transplanting the gut microbiota of AD patients into germ-free mice exacerbates AD pathology [87]. Currently, improving liver function and regulating the gut microbiota are popular topics in AD treatment research. The drug sodium oligomannate, which regulates the gut microbiota, has entered phase III clinical trials, and its ability to improve cognitive function in AD patients has been demonstrated [88]. With further research into the brain–liver–gut axis, more methods for treating AD patients are expected to emerge in the future.

#### Parkinson’s disease

PD is the second most common type among all degenerative diseases of the central nervous system [89]. The Lewy body is one of the important features of PD, as it is mainly composed of misfolded alpha synuclein (alpha Syn), PD is classified as synucleinopathy [90]. The clinical manifestations of PD include motor symptoms such as static tremor and bradykinesia, as well as non motor symptoms such as hypotension, memory loss, and urinary dysfunction [90]. Age is the most important risk factor for PD. Other potential influences, such as excessive physical activity or head trauma, liver dysfunction, gut microbiota dysbiosis, smoking, coffee, and toxin exposure, also play roles in the pathogenesis of PD [91–93]. Although the exact cause of PD remains unknown, findings from recent studies indicate that signals from the gut significantly contribute to PD progression [94]. Animal experiments in which the gut microbiota from PD patients was transplanted into mice revealed increased αSyn expression in mice receiving the transplants, which in turn led to neuroinflammation and motor symptoms [95]. Another animal study demonstrated that transplanting the gut microbiota from normal mice into PD mice alleviated BBB damage, inhibited neuroinflammation in the SN, and suppressed the TLR4/MyD88/NF-κB signaling pathway and downstream proinflammatory products in both the SN and colon, thereby alleviating PD symptoms in mice [96]. In addition to gut-derived signals, the liver is also closely associated with PD development. For example, a recent clinical study revealed that liver fibrosis leads to significant cognitive decline in PD patients [97]. On the other hand, the liver produces hepatocyte growth factor (HGF), which has been shown to increase the growth of nigrostriatal dopaminergic axonal terminals; promote the growth and differentiation of neuronal stem cells; reduce dopaminergic neuron loss in the SN of PD animals; and exert anti-inflammatory and antioxidant effects by modulating microglia, TNF-α, and extracellular signal-regulated kinase 1/2 (ERK1/2) [98]. Therefore, maintaining a stable gut microbiota and healthy liver function may be crucial directions for treating PD.

#### Chronic traumatic encephalopathy

TBI is a major cause of death and disability worldwide [99]. In recent years, it has been increasingly recognized that TBI not only is a short-term, acute injury but also can trigger psychiatric symptoms and neurodegenerative diseases, such as depression, anxiety, memory decline, and cognitive impairment [100], referred to as CTE. These chronic effects are not only found in severe TBI patients but are also gradually being discovered in patients previously classified as having moderate or mild TBI [101]. The symptoms and impact extend far beyond the initial injury. A prospective cohort study evaluating long-term social cognition after mild TBI (mTBI) indicated that even mTBI can affect overall function [102]. Younger patients are more likely to develop psychiatric symptoms such as depression and anxiety, whereas older patients are prone to symptoms such as fatigue, balance issues, memory decline, and reduced attention [103]. The exact mechanisms underlying CTE development are not fully understood but are currently thought to involve chronic inflammation, neurotransmitter metabolism abnormalities, BBB damage, and abnormal protein deposition [104–106]. The activity of the brain–liver–gut axis may contribute to the persistence of these factors, possibly being an important cause of CTE. The temporary, regular inflammatory response following primary injury after TBI is generally beneficial [107]. Some inflammatory factors can regulate neural repair, such as IL-4 or low-dose IFNγ, which can induce M2-like microglia to release neurotrophic factors and promote neurogenesis [108]. However, prolonged, persistent neuroinflammation can impede neural recovery; for example, LPS, TNF, IL-1β, and IL-6 can stimulate M1 microglia, reducing hippocampal neurogenesis in adult mice [109, 110]. The increase of these inflammatory factors is intricately linked to the disruption of gut microbiota and liver immune activation. Numerous studies have confirmed that persistent inflammatory responses following TBI contribute to subsequent neurodegenerative disease progression [111, 112]. Additionally, increased levels of glutamate and other excitatory neurotransmitters in the cerebrospinal fluid of TBI patients are correlated with gut microbiota dysbiosis, which can activate neuronal glutamate receptors, causing excitotoxic neuronal injury [113]. TBI-induced damage to the BBB and axonal injuries lead to cerebral hypoperfusion and the formation of pathological proteins, such as Aβ and hyperphosphorylated tau protein; Aβ oligomers further induce vascular injury; therefore, a vicious cycle is formed [114]. In vivo positron emission tomography (PET) studies suggest a correlation between past TBI and tau and Aβ tracer uptake [115]. MRI diffusion imaging after subacute, one-year, and five-year periods following trauma reveals U-shaped trajectories of white matter-related changes (initial recovery followed by decline), which may help explain the relationship between initial recovery and long-term symptoms [116]. The liver is a key organ in Aβ metabolism. Findings from animal experiments have indicated that improving liver function can reduce Aβ accumulation in the brains of TBI mice and promote recovery [23]. Findings from previous studies have suggested that the brain–liver–gut axis has made significant contributions to CTE; therefore, targeting this axis could be crucial in optimizing CTE treatment.

#### Depression

**Figure 2:**
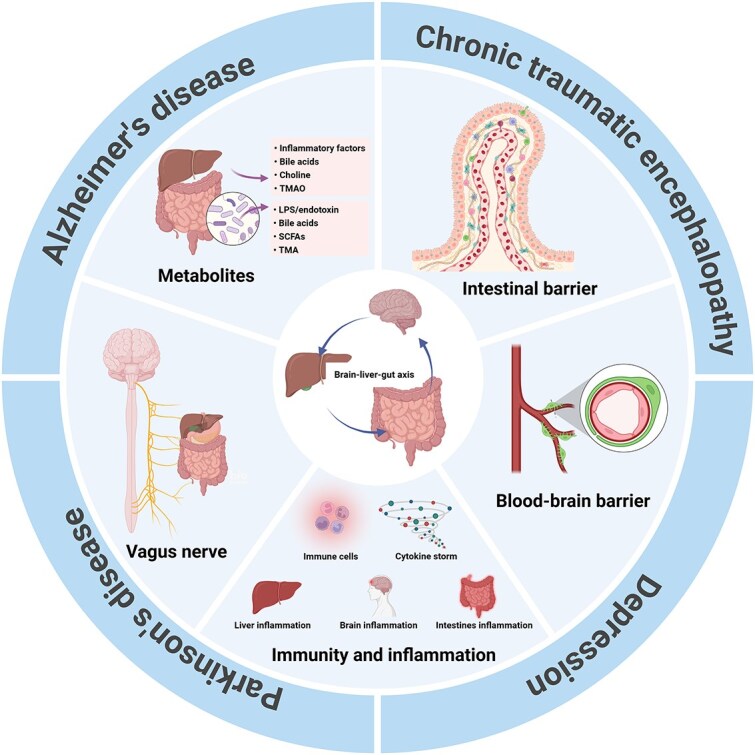
An outline map of the various roles of the brain-liver-gut axis in neurological diseases, including Alzheimer’s disease, Parkinson’s disease, chronic traumatic encephalopathy and depression that are currently known. Created with BioRender.com

The disease burden caused by depression is widely known [117]. In addition to psychological factors, depression is also highly linked to neurotransmitter imbalances, neuroinflammation, increased BBB permeability, neurodevelopmental deficits, and synaptic dysfunctions [118]. The liver contributes to depression development by regulating BAs, inflammatory factors, and toxic substances such as ammonia and manganese. In hyperammonemia, astrocytes and microglia produce elevated levels of the proinflammatory cytokines IL-1β and IL-6. These cytokines and activated microglia in the hippocampus may be associated with depression-like behavior [119]. The gut microbiota is intricately linked to depression through a variety of mechanisms, BA metabolism, inducing neuroinflammation, affecting neurotransmitter levels, and stimulating vagal signaling. Transferring gut microbiota from depressed patients can induce depression-like behavior in antibiotic-pretreated rats, whereas fecal microbiota transplantation (FMT) can alleviate depression symptoms in mice subjected to chronic social defeat stress [120]. Both the liver and the gut microbiota are involved in BA metabolism, and elevated levels of circulating BAs may synergistically lead to the loss of BBB integrity, resulting in depressed and anxious psychopathologies [121]. Recent research findings have confirmed that the gut microbiota and BA are dysregulated in patients with depression [122]. Gut microbes are vital for the metabolism of SCFAs, which participate in neuroinflammation and influence the levels of many neurotransmitters. Clinical and animal studies have shown that low SCFA levels are associated with depression, with reduced SCFA levels found in the feces of depression patients [123]. Additionally, the loss of gut barrier integrity and translocation of gut microbes can trigger a TLR-mediated proinflammatory cascade. TLR activation can polarize microglia to the M1 phenotype, stimulating brain inflammation and leading to depression symptoms [124] (Figure 2).

### Potential treatments

#### Antibiotics

Generally, antibiotics can clear the gut of bacteria, thereby reducing bacterial translocation, decreasing the liver burden, and alleviating neuroinflammation, thus providing therapeutic effects. For example, rifaximin, an unabsorbable, broad-spectrum, gastrointestinal-specific antibiotic, lowers serum ammonia and endotoxin levels, potentially alleviating symptoms of irritable bowel syndrome and nonalcoholic fatty liver disease while improving cognitive function in HE patients [125–127]. Moreover, evidence suggests that rifaximin can reduce motor fluctuations in PD patients without affecting levodopa treatment [128]. In addition to combating dysbiosis, certain antibiotics, such as minocycline and doxycycline, have been shown to inhibit matrix metalloproteinase activity and prevent mitochondrial dysfunction, microglial activation, and αSyn aggregation, offering therapeutic benefits for PD and AD and improving long-term outcomes in TBI patients [129–131]. Despite these beneficial effects, the use of antibiotics remains controversial, with evidence indicating that long-term use can eliminate beneficial bacteria, leading to dysbiosis and disease exacerbation [131]. Additionally, increased resistance and side effects limit their use. Therefore, combining traditional antibiotics with novel nanomaterials to improve the anti-inflammatory effects while minimizing antibacterial actions may offer a promising therapeutic approach [132].

#### Probiotics and prebiotics

Probiotics are believed to hold promise in modulating and mitigating cardio-cerebral diseases [133]. Commonly used probiotics in research include Lactobacillus, Streptococcus, and Bifidobacterium, which significantly ameliorate symptoms of liver- and brain-related diseases such as nonalcoholic fatty liver disease, PD, depression, and epilepsy [134]. have also been shown to enhance cognitive function in individuals with AD [135]. Studies have shown that transgenic AD mice treated with probiotics have improved cognitive ability and reduced hippocampal Aβ plaque formation [136]. Mechanistically, probiotics improve microbiota structure, influence metabolic products, tighten junctions to alleviate endotoxemia, and suppress systemic immune dysfunction [137]. A prebiotic, as defined by the International Scientific Association for Probiotics and Prebiotics, is ‘a substrate that is selectively utilized by host microorganisms, conferring a health benefit’ [138]. Many indigestible dietary fibers, carbohydrates, oligosaccharides, and polyphenols act as prebiotics. Beneficial bacteria in the gut metabolize prebiotics, increasing the production of SCFAs such as acetate, propionate, and butyrate, which participate in neuroregulatory processes, anti-inflammatory processes, and gut barrier and BBB regulation, thereby alleviating PD symptoms [139]. Findings from other studies have indicated that oral prebiotics can reduce plaque formation, oxidative stress, and inflammation, thereby improving memory and learning in AD model rats and mice [140]. Despite positive experimental data, there is a lack of convincing human trial data, and there is a need for a better understanding of the mechanisms involved. Future research may provide sufficient evidence to validate these therapies.

#### Fecal microbiota transplantation

FMT is a therapeutic method that collects the gut microbiota of healthy donors and introduces it into patients to restore the microbiota composition to a healthy state. Animal studies have demonstrated positive effects of FMT on PD, showing that FMT can restore the gut microbiota balance and inhibit LPS–TLR4-induced inflammation in rotenone-induced PD models [96]. Additionally, gut dysbiosis in AD mice induces intestinal inflammation, impairing neurogenesis and leading to memory decline, whereas FMT can improve spatial learning and memory in AD mice [141]. FMT also has beneficial effects in treating inflammatory anxiety and depression, normalizing the gut microbiota and reducing the levels of inflammatory factors in mice with depression- and anxiety-like behaviors [142]. Furthermore, FMT reduces brain ventricle edema and reverses behavioral abnormalities after TBI by decreasing ionized calcium-binding adaptor molecule 1 [143]. However, issues such as effectiveness and safety concerns regarding pathogen transfer are still faced in FMT, and further research is necessary to increase FMT efficacy and minimize its adverse effects.

#### Dietary interventions

Nutritional factors play a significant role in neurodegenerative diseases, potentially by altering the gut microbiota composition or metabolism. For example, the Mediterranean diet (MD), some studies suggest that MD can reduce the risk of neurodegenerative diseases such as PD and AD [144]. This effect is hypothesized to be due to high dietary fiber altering the gut microbiota and reducing neuroinflammation [145]. Additionally, omega-3 polyunsaturated fatty acids (PUFAs) directly participate in neuroinflammation and hormonal regulation [146]. Dietary supplementation with PUFAs can alleviate symptoms and depression in PD and AD patients [147]. Conversely, a high-cholesterol Western diet may induce depression-like symptoms, whereas high fruit and vegetable intake reduces the incidence of depression [148]. Overall, dietary interventions, as adjunctive treatments, offer significant therapeutic potential for chronic neurological diseases.

#### Vagus nerve stimulation

The vagus nerve directly connects the brain with the liver and gut. Modulating vagus nerve signals can mediate therapeutic effects. Studies have shown that rats subjected to vagotomy do not exhibit behaviors indicative of illness after LPS injection. Additionally, vagus nerve stimulation (VNS) has been demonstrated to be an effective therapy for reducing the inflammation induced by endotoxemia [149], and VNS has been shown to decrease gut permeability and improve BBB integrity [150]. Early preclinical study findings have indicated that VNS treatment after head injury can improve motor and cognitive outcomes while reducing secondary neuronal damage [151]. Currently, VNS has been widely used in treat depression, epilepsy and primary headaches, with evidence supporting its beneficial effects for PD, AD, anxiety, and TBI. However, the precise mechanisms of action of VNS therapy remain elusive, and more comprehensive research is required to fully understand its therapeutic potential and broaden its clinical applications.

## Conclusions

With a deeper understanding of the the brain–liver–gut axis and gut microbiota, maintaining a stable balance of gut, liver, and brain functions can alleviate symptoms of various diseases, whereas disruption of these functions can lead to various diseases, especially CNS diseases. The aim of this review was to elucidate the interactions in the complex network of the brain–liver–gut axis and to discuss the pathogenesis of neurological disorders such as AD, PD, CTE, and depression. Currently, seveal treatments based on the brain-liver-gut axis have been proven by preclinical studies and randomized controlled trials. As the understanding of the mechanisms of brain–liver–gut axis function continues to grow, future research may uncover safer and more effective treatments based on this axis.

## Data Availability

Not applicable.
